# Downregulated miR-98-5p promotes PDAC proliferation and metastasis by reversely regulating MAP4K4

**DOI:** 10.1186/s13046-018-0807-2

**Published:** 2018-07-03

**Authors:** Yue Fu, Xinchun Liu, Qiuyang Chen, Tongtai Liu, Cheng Lu, Jun Yu, Yi Miao, Jishu Wei

**Affiliations:** 1 Pancreas Center, the First Affiliated Hospital of Nanjing Medical University, 300 Guangzhou Road, Nanjing, Jiangsu Province, People’s Republic of China; 2 Department of General Surgery, The Affiliated Changzhou NO.2 People’s Hospital With Nanjing Medical University, 68 Gehu Road, Changzhou, Jiangsu Province, People’s Republic of China; 3 Department of Surgery, Johns Hopkins Medical Institutions, 600 N Wolfe Street, Baltimore, MD USA

**Keywords:** MiR-98-5p, MAP4K4, Proliferation, Migration, Invasion, MAPK/ERK signaling pathway, Pancreatic ductal adenocarcinoma

## Abstract

**Background:**

The aberrant expression of microRNAs (miRNAs) has emerged as important hallmarks of cancer. However, the molecular mechanisms underlying the differences of miRNA expression remain unclear. Many studies have reported that miR-98-5p plays vital functions in the development and progression of multiple cancers. However, its role in pancreatic ductal adenocarcinoma (PDAC) remains unknown.

**Methods:**

The expression of miR-98-5p and its specific target gene were determined in human PDAC specimens and cell lines by miRNA qRT-PCR, qRT-PCR and western blot. The effects of miR-98-5p depletion or ectopic expression on PDAC proliferation, migration and invasion were evaluated in vitro using CCK-8 proliferation assays, colony formation assays, wound healing assays and transwell assays. Furthermore, the in vivo effects were investigated using the mouse subcutaneous xenotransplantation and pancreatic tail xenotransplantation models. Luciferase reporter assays were employed to identify interactions between miR-98-5p and its specific target gene.

**Results:**

MiR-98-5p expression was significantly lower in cancerous tissues and associated with tumor size, TNM stage, lymph node metastasis and survival. Notably, a series of gain- and loss-of-function assays elucidated that miR-98-5p suppressed PDAC cell proliferation, migration and invasion both in vitro and in vivo. Luciferase reporter assays, western blot and qRT-PCR revealed MAP4K4 to be a direct target of miR-98-5p. The effects of ectopic miR-98-5p were rescued by MAP4K4 overexpression. In contrast, the effects of miR-98-5p depletion were impaired by MAP4K4 knockdown. Furthermore, miR-98-5p suppressed the MAPK/ERK signaling pathway through downregulation of MAP4K4. In addition, the expression level of miR-98-5p was negatively correlated with MAP4K4 expression in PDAC tissues and cell lines.

**Conclusions:**

These results suggest that downregulation of miR-98-5p promotes tumor development by downregulation of MAP4K4 and inhibition of the downstream MAPK/ERK signaling, thus, highlighting the potential of miR-98-5p as a therapeutic target for PDAC.

**Electronic supplementary material:**

The online version of this article (10.1186/s13046-018-0807-2) contains supplementary material, which is available to authorized users.

## Background

Pancreatic ductal adenocarcinoma (PDAC), which accounts for 95% of all pancreatic cancer cases, is an aggressive digestive cancer that is highly chemoresistant, proliferative and anti-apoptotic with a dismal 5 year survival rate of less than 8% [1]. Due to lack of characteristic symptoms and effective methods for the early detection of PDAC, over 80% of patients present too late for curative management [2, 3]. Therefore, identification of novel and effective therapeutic target for PDAC is an urgent issue.

MicroRNA (miRNA), an endogenous, small non-coding RNA molecule of 19–22 nucleotides in length, downregulates the expression of target gene by binding to the 3′-untranslational regions (3’-UTR) of specific messenger RNAs (mRNAs) [4, 5]. MiRNAs control a wide range of cellular processes, such as cell proliferation, apoptosis, metastasis and survival [5]. Aberrantly expressed miRNAs have been reported to serve as tumor suppressors or promoters in various human cancers [6–9]. Moreover, emerging reports confirmed that miR-98-5p was downregulated and correlated with malignant progression in certain carcinoma, including HCC, ovarian cancer, colon cancer and lung cancer [10–13]. However, the role of miR-98-5p in PDAC remained to be elucidated. In this study, we discover that miR-98-5p is downregulated in PDAC and that miR-98-5p overexpression suppresses the progression of PDAC both in vitro and in vivo.

Mitogen-activated protein 4 kinase 4 (MAP4K4, also called hepatocyte progenitor kinase-like/germinal center kinase-like kinase), a serine/threonine kinase belongs to the mammalian STE20/MAP4K family, was involved in several biological processes, such as cell motility, rearrangement of the cytoskeleton and cell proliferation [14–16]. Overexpression of MAP4K4 was identified as a prognostic indicator in multiple cancers, including hepatocellular carcinoma (HCC), PDAC, lung cancer, colorectal cancer (CRC) and prostate cancer [17–21]. Furthermore, MAP4K4 knockdown was found to induce apoptosis, cell cycle arrest, and inhibition of cell proliferation, migration and invasion in different cancer cells [18, 22–24]. The findings of present study suggest that MAP4K4 is a putative direct target of miR-98-5p.

Therefore, we investigate the role of miR-98-5p in PDAC and its relationship with MAP4K4. Our findings suggest that miR-98-5p downregulation was vital for the progression of PDAC, highlighting the potential of miR-98-5p as a therapeutic target in PDAC.

## Methods

### Patients and samples

MiR-98-5p and MAP4K4 mRNA expression for 178 pancreatic cancer patients are downloaded from The Cancer Genome Atlas (TCGA). The Gene Expression Omnibus (GEO; GSE62452, GSE15471, GSE62165) data are used to analyze the expression of MAP4K4 in PDAC tissues and matched non-cancerous or normal pancreas tissues. Briefly, GSE62452 includes 69 cancerous tissues and 61 paired non-cancerous tissues, GSE15471 includes 39 pairs of cancerous and non-cancerous tissues. GSE62452 includes 118 cancerous tissues and 13 normal pancreas tissues. In addition, 52 pairs of pancreatic specimens were obtained from patients who underwent surgical resection for PDAC at Pancreas Centre of the First Affiliated Hospital of Nanjing Medical University from June 2015 to September 2016. No chemotherapy or radiation therapy was administered before tumor excision. Both cancerous and corresponding non-cancerous tissues were immediately stocked at liquid nitrogen after surgical removal and stored at − 80 °C until processed. The diagnosis of PDAC was validated by two individual pathologists according to the World Health Organization classification criteria. Written informed consents were obtained from all patients undergoing surgery. The Ethics Committees of the First Affiliated Hospital of Nanjing Medical University approved the study.

### Reagents

LV2-hsa-miR-98-5p-mimics, LV2-hsa-miR-98-5p-inhibitor and their corresponding control lentivirus were constructed and synthesized by GenePharm (Shanghai, China). MAP4K4 overexpression plasmid (Lv-MAP4K4) and its specific small interference RNA (Si-MAP4K4) were designed and synthesized from GENEWIZ (Soochow, China). Anti-human MAP4K4 antibody was obtained from Abcam (BRISTOL, UK). Anti-human p38, p-p38, ERK, p-ERK, GAPDH antibodies were purchased from Cell Signaling Technology (Danvers, MA, USA). The MAPK inhibitor SB203580 was purchased from Cell Signaling Technology (Danvers, MA, USA). Cell counting kit-8 (CCK-8) reagent was obtained from Dojindo (Kumamoto, Japan). TRIzol reagent and PrimeScript RT Master Mix were obtained from Takara (Shiga, Japan). FastStart Universal SYBR Green Master was purchased from Roche.

### Cell culture and transfection

The human PDAC cell lines (SW1990, MIAPACA-2, BXPC-3, CFPAC-1, COLO357 and PANC-1) and human pancreatic duct epithelial cell line HPNE were obtained from the Cell Bank of Chinese Academy of Science (Shanghai, China). All cell lines were cultured with Dulbecco’s Modified Eagle’s Medium (DMEM) supplemented with 10% fetal bovine serum (FBS) and 100 μL/mL penicillin/streptomycin in a humidified incubator containing 5% CO_2_ at 37 °C.

LV2-hsa-miR-98-5p-mimics, LV2-hsa-miR-98-5p-inhibitor and their corresponding control lentivirus were initially proved by DNA sequencing before transfection. All lentivirus were used to infect CFPAC-1 and MIAPACA-2 cells with an appropriate multiplicity of infection (MOI) respectively. The stable overexpression or knockdown cell lines were generated by selecting transfected cells in complete culture medium containing puromycin (5 μg/mL) for at least 5 days. In addition, Lv-MAP4K4 plasmid and specific Si-MAP4K4 were used to upregulate or silence MAP4K4 expression in PDAC cell lines by Lipofectamine 3000 (Invitrogen, USA) according to the manufacturer’s protocols.

### RNA extraction, quantitative real-time PCR (qRT-PCR) and miRNA qRT-PCR

Total RNA was extracted from tissues and cells with the TRIzol reagent. For mRNA quantitative assay, total RNA was reversely transcribed into cDNA using PrimeScript RT Reagent. The process of qRT-PCR amplification was performed using the Step One Plus Real-Time PCR System (Applied Biosystems, Carlsbad, CA, USA) with FastStart Universal SYBR Green Master. Glyceraldehyde-3-phosphate dehydrogenase (GAPDH) was used as the internal control. For miRNA quantitative assay, target specific reverse transcription and TaqMan miRNA assays were carried out using the Hairpin-it™ miRNA qPCR Quantitation Kit (GenePharma, China). The reactions were also performed by the Step One Plus Real-Time PCR System. The snRNA U6 was served as the internal control. All assays mentioned were performed according to the relevant manufacturer’s protocols. The levels of gene expression were calculated using the 2^-ΔΔCT^ methods. All experiments were repeated at least three times. All primers were showed in Additional file 1: Table S1.

### Western blot

Total protein were extracted from tissues and cells using the protein extraction kit (Beyotime, China). Protein concentrations were measured using the BCA Protein Assay Kit (Pierce Chemical, USA). The proteins were resolved by sodium dodecyl sulfate-polyacrylamide gel electrophoresis (SDS-PAGE) and transferred to a polyvinylidene difluoride (PVDF) membrane. After blocking with 4% non-fat milk in Tris-buffered saline at room temperature for 2 h, membranes were incubated at 4 °C overnight with corresponding primary antibodies. The membranes were incubated with HRP-conjugated anti-mouse or anti-rabbit IgG at room temperature for 2 h and then washed with TBST buffer three times. Protein expression levels were visualized using the ECL Western Blot Kit (Pierce Chemical, USA). The integrated density of protein bands was quantified using Image Lab software (Bio-Rad, USA). GAPDH was used as an internal control.

### CCK-8 assay

Different groups of cells were plated at 96-well plate with a density of 2 × 10^3^ cells per well, After culture for 24 h, 110 μL complete medium containing 10 μL CCK-8 reagents were added to respective wells at different time points (0 h, 24 h, 48 h, 72 h and 96 h). The plates were incubated in dark at 37 °C for 2 h and then analyzed at 450 nm absorbance. At least five wells were assessed for each group.

### Colony formation assay

Different groups of cells were seeded at 6-well plates with a density of 6 × 10^2^ cells per well. The cells were stained with 0.5% crystal violet after culture for 2 weeks. The culture medium was changed every 2 days. Finally, the number of clones was counted to evaluate cell proliferation.

### Cell migration and invasion assay

Cell migration and invasion was assessed using transwell filters purchased from BD Biosciences (Franklin Lakes, NJ). After culture in serum-free medium for 24 h, cells were seeded into the upper chamber (2.5 × 10^4^/well) containing 200 μL serum-free medium with an uncoated or Matrigel-coated membrane. 700 μL complete medium was added to the lower chamber. Following incubation for 48 h at 37 °C in a humidified 5% CO_2_ incubator, cells on the upper surface of the filters were removed with a cotton swab. After staining with 0.5% crystal violet, cells migrated or invaded into the lower surface were photographed and counted in 5 random fields under a microscope (magnification, 100 ×).

For wound healing assays, different groups of cells (1 × 10^6^) were seeded in 6-well plate and were allowed to grow to 80–90% confluence respectively. The linear scratch wounds (in triplicate) were created by 200 μL pipette tip. Following washing by PBS for 2–3 times, the cells were maintained in DMEM containing 1% FBS in a humidified incubator under 5% CO_2_ at 37 °C. Images were photographed at same areas and the wound healing was measured at 0 h, 24 h and 48 h. Image J Plus was used to quantify the wound healing assay.

### Luciferase reporter assay

Sequences corresponding to the 3’-UTR of MAP4K4 mRNA and containing the wild-type or mutated miR-98-5p binding sequence were synthesized by GeneScript (Nanjing, China). The sequences of MAP4K4 3’-UTR reporter constructs (MAP4K4-WT and MAP4K4-MUT) were cloned into the FseI and XbaI restriction sites of the pGL3 luciferase control reporter vector (Promega, USA). CFPAC-1 and MIAPACA-2 cells were seeded in 24-well plates (5 × 10^5^ per well) and were incubated for 24 h before transfection. Furthermore, CFPAC-1 cells were co-transfected with either MAP4K4-WT or MAP4K4-MUT reporter plasmids combined with miR-98-5p-mimics or its control oligoribonucleotides. MIAPACA-2 cells were co-transfected with either MAP4K4-WT or MAP4K4-MUT reporter plasmids together with miR-98-5p-inhibitor or its negative control oligoribonucleotides using lipofectamine 3000 (Invitrogen). In addition, CFPAC-1 and MIAPACA-2 cells were transfected with Renilla luciferase expression plasmid as a reference control. Firefly and Renilla luciferase activities were detected using dual luciferase reporter assays (Promega, E1910, WI, USA) at 48 h post-transfection according to the manufacturer’s instruction. The relative luciferase activity was calculated according to the ratio of firefly fluorescence and Renilla fluorescence.

### In vivo tumor growth and metastasis assay

All animal assays were performed according to the institutional guidelines of Nanjing Medical University and Use Committee. All female BALB/c nude mice aged 4 weeks were purchased from Model Animal Research Center of Nanjing University. For the subcutaneous implant model, cells (1 × 10^6^ cells per flank, 100 μL per flank) stably transfected with miR-98-5p-mimics, miR-98-5p-inhibitor or corresponding controls were injected subcutaneously into the flank region of nude mice respectively. Tumor measurements were taken with calipers every 4 days. All mice were sacrificed after 4 weeks. Tumor volume was determined using the formula: 0.44 × length × width^2^. For the pancreatic tail metastasis model, cells (5 × 10^5^ cells per mice, 50 μL per mice) stably transfected with miR-98-5p-mimics, miR-98-5p-inhibitor or corresponding controls were separately injected into the pancreatic tail of nude mice orthotopically. All mice were sacrificed after inoculation of 6 weeks, and the liver metastatic nodules were examined by counted.

### Statistical analysis

All data were analyzed using the SPSS software (version 17.0) and GraphPad Prism software (Version 5.0). The data were expressed as mean ± standard deviation (SD). Clinicopathologic findings with miR-98-5p and MAP4K4 were compared by Pearson χ2 test. The paired Student’s *t*-test was applied to compare miR-98-5p and MAP4K4 expression in cancerous and paired non-cancerous tissues. Survival analysis was measured by Kaplan Meier and Log-rank test. The comparisons between two groups were done by independent Student’s *t*-test. Differences were considered statistically significant at *P* < 0.05.

## Results

### MiR-98-5p is downregulated in PDAC tissues and cell lines

**Fig. 1:**
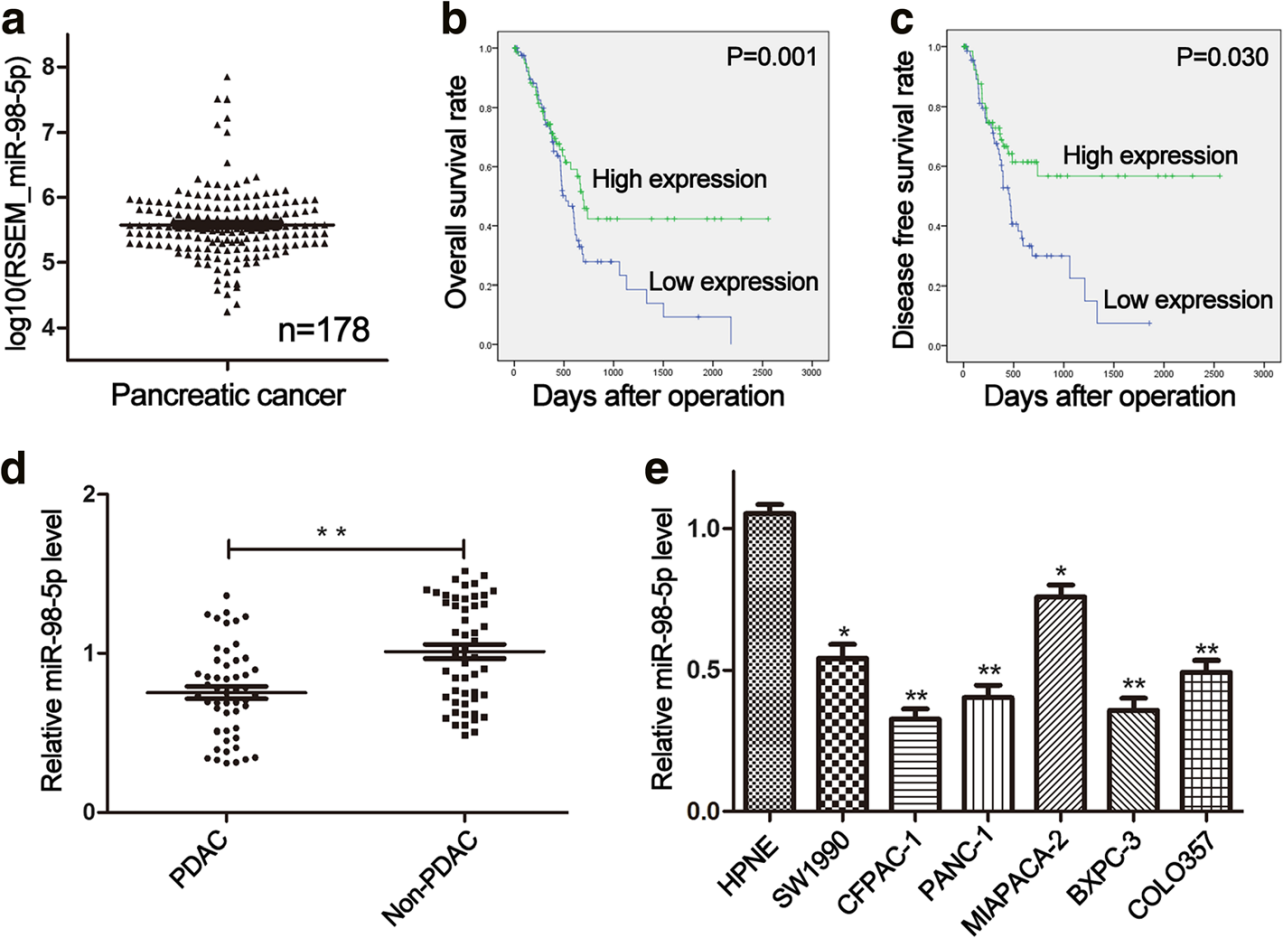
miR-98-5p is downregulated in PDAC tissues and cell lines. **a** MiR-98-5p expression in 178 PDAC tissues obtained from TCGA was shown. **b, c** Downregulation of MAP4K4 was associated with poorer prognosis of PDAC. **d** Lower expression of miR-98-5p was found in PDAC tissues than that in non-cancerous tissues by miRNA qRT-PCR. **e** The expression of miR-98-5p in different PDAC cell lines was shown. *P < 0.05, ** *P* < 0.01. The data expressed as the mean ± SD. All experiments were repeated at least three times

According to the data obtained from TCGA, miR-98-5p was generally expressed in PDAC tissues (*n* = 178) (Fig. 1a). The expression level of miR-98-5p detected by miRNA qRT-PCR was significantly decreased in 52 cancerous tissues compared with paired non-cancerous tissues (Fig. 1d). Consistently, miR-98-5p was also significantly downregulated in PDAC cell lines relative to normal HPNE (Fig. 1e). The association between miR-98-5p expression and clinicopathological characteristics was depicted in Table 1. Low levels of miR-98-5p were significantly associated with some indicators of PDAC progression, such as tumor size, TNM stage and lymph node metastasis. By dividing all patients from TCGA into low and high expression group based on the median value, survival analysis suggested that lower expression of miR-98-5p could have a shorter overall survival and disease-free survival rate in PDAC (Fig. 1b-c). These results revealed that miR-98-5p was downregulated in PDAC.

**Table 1:** The clinicopathological relevance analysis of miR-98-5p and MAP4K4 expression in PDAC patients

Characteristic	miR-98-5p			MAP4K4		
	Low	High	*p* value	Low	High	*p* value
All cases	25	27		26	26	
Age						
< 65	15	14	0.554	16	13	0.402
≥ 65	10	13		10	13	
Gender						
Male	13	15	0.797	15	13	0.578
Female	12	12		11	13	
CA199						
≤ 100	9	15	0.158	15	9	0.095
> 100	16	12		11	17	
Tumor size						
≤ 2.5 cm	8	20	0.002*	19	9	0.005*
> 2.5 cm	17	7		7	17	
Differentiation grade						
Well-moderately	7	14	0.080	13	8	0.158
Poorly	18	13		13	18	
TNM						
I-IIA	10	19	0.028*	20	9	0.002*
IIB-IV	15	8		6	17	
Lymph node metastasis						
Absence	9	21	0.002*	19	11	0.025*
Presence	16	6		7	15	
Blood vessel invasion						
Absence	10	18	0.054	17	11	0.095
Presence	15	9		9	15	

*p < 0.05 Statistical significant difference

### MiR-98-5p inhibits the proliferation of PDAC cells

**Fig. 2:**
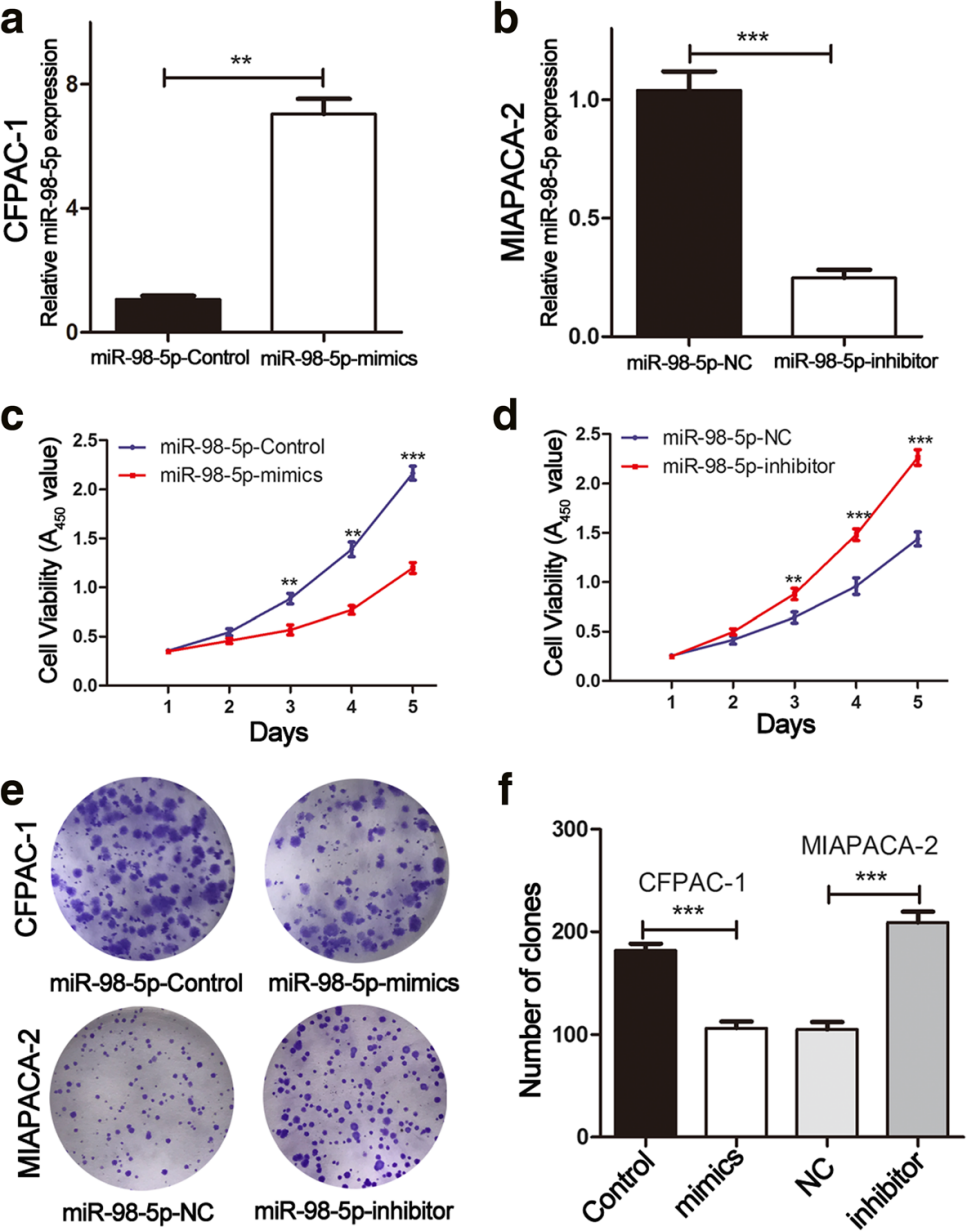
miR-98-5p suppresses PDAC cell proliferation. **a, b** MiRNA qRT-PCR was used to verify the expression of miR-98-5p in cells transfected with miR-98-5p-mimics and miR-98-5p-inhibitor respectively. **c, d** CCK-8 was used to detect the proliferation of cells transfected with miR-98-5p-mimics and miR-98-5p-inhibitor. **e, f** Effects of miR-98-5p alteration on the colony formation of PDAC cells. **P < 0.01, ***P < 0.001. The data expressed as the mean ± SD. All experiments were repeated at least three times

To investigate the role of miR-98-5p, CFPAC-1 and MIAPACA-2 cells were selected for transfection based on the miR-98-5p expression level in PDAC cells (Fig. 1e). The efficiency of transfection of the cell lines (CFPAC-1-control, CFPAC-1-mimics, MIAPACA-2-NC and MIAPACA-2-inhibitor) was validated by miRNA qRT-PCR. As shown in Fig. 2a-b, miR-98-5p was significantly upregulated in CFPAC-1-mimics and downregulated in MIAPACA-2-inhibitor compared with the levels measured in the control groups.

Since miR-98-5p expression was significantly associated with tumor size, we further investigated whether miR-98-5p could affect PDAC cell proliferation. The influence of miR-98-5p on PDAC cell proliferation was investigated using CCK-8 and colony formation assays. The proliferation rate of CFPAC-1 cells transfected with miR-98-5p-mimic was significantly decreased relative to that of the control group (Fig. 2c). In contrast, MIAPACA-2 cells transfected with the miR-98-5p-inhibitor exhibited a significant increase in proliferation compared with that of the control group (Fig. 2d). As shown in Fig. 2e-f, miR-98-5p overexpression impaired colony formation ability, whereas miR-98-5p depletion had the opposite effects. These results revealed that miR-98-5p inhibited PDAC cell proliferation in vitro.

### MiR-98-5p suppresses the migration and invasion of PDAC cells

**Fig. 3:**
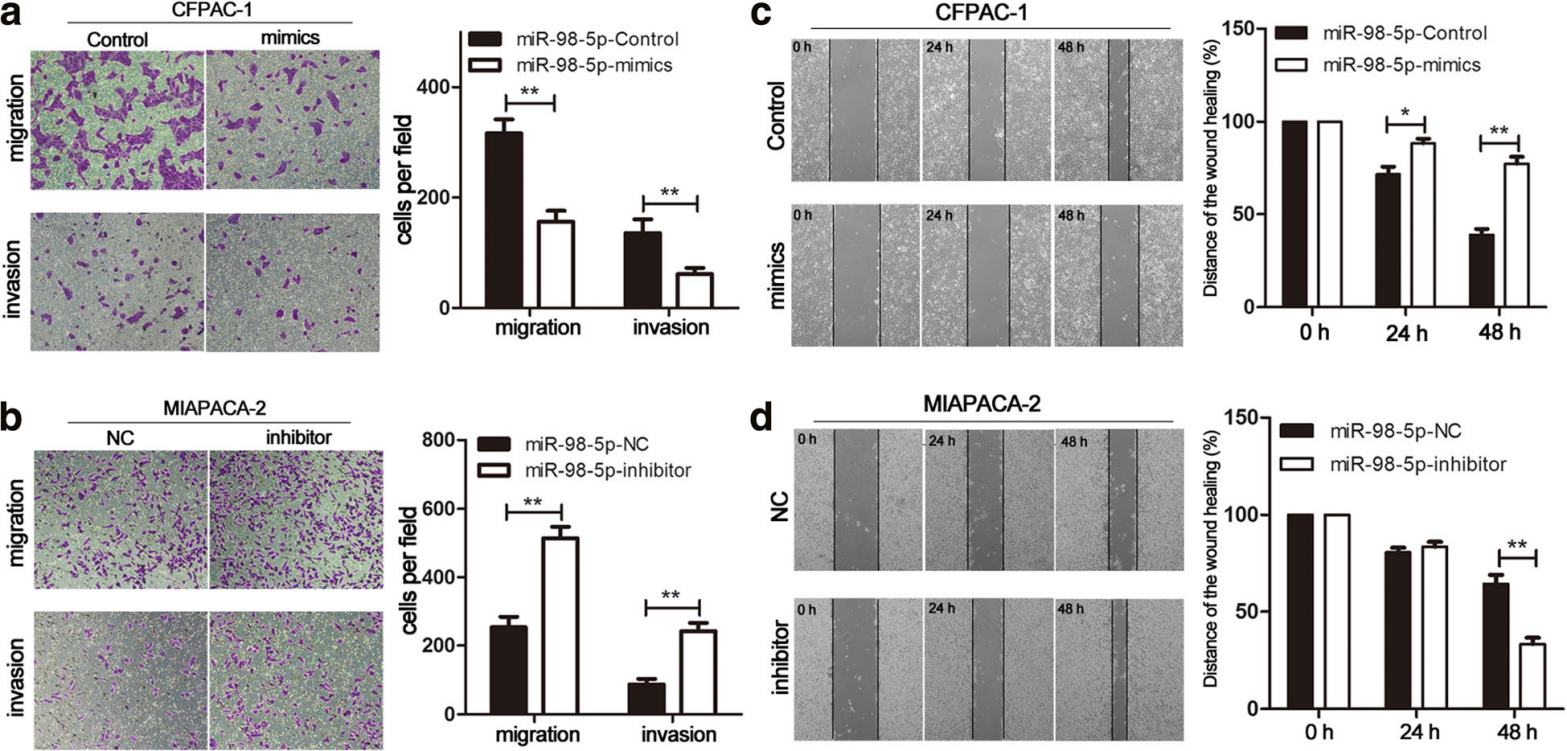
miR-98-5p inhibits PDAC cell migration and invasion. **a, b** The migration and invasion of PDAC cells transfected with miR-98-5p-mimics or miR-98-5p-inhibitor were determined by transwell assays (magnification, 100 ×). **c, d** Effects of miR-98-5p alteration on the wound healing ability of PDAC cells. Relative ratio of wound closure per field was shown. *P < 0.05, **P < 0.01, ***P < 0.001. The data expressed as the mean ± SD. The data came from at least three independent experiments

Since lower miR-98-5p level was significantly associated with TNM stage and lymph node metastasis, we speculated that miR-98-5p might suppress the migration and invasion of PDAC cells. Here the influence of miR-98-5p on PDAC cell migration and invasion was detected using transwell and wound healing assays. The migration and invasion of PDAC cells was significantly decreased following overexpression of miR-98-5p (Fig. 3a). Conversely, miR-98-5p depletion significantly promoted PDAC cell migration and invasion (Fig. 3b). As shown in Fig. 3c-d, data derived from wound healing assays also showed the same results. These findings suggested that miR-98-5p suppressed the migration and invasion of PDAC cells.

### MAP4K4 is a direct target of miR-98-5p

**Fig. 4:**
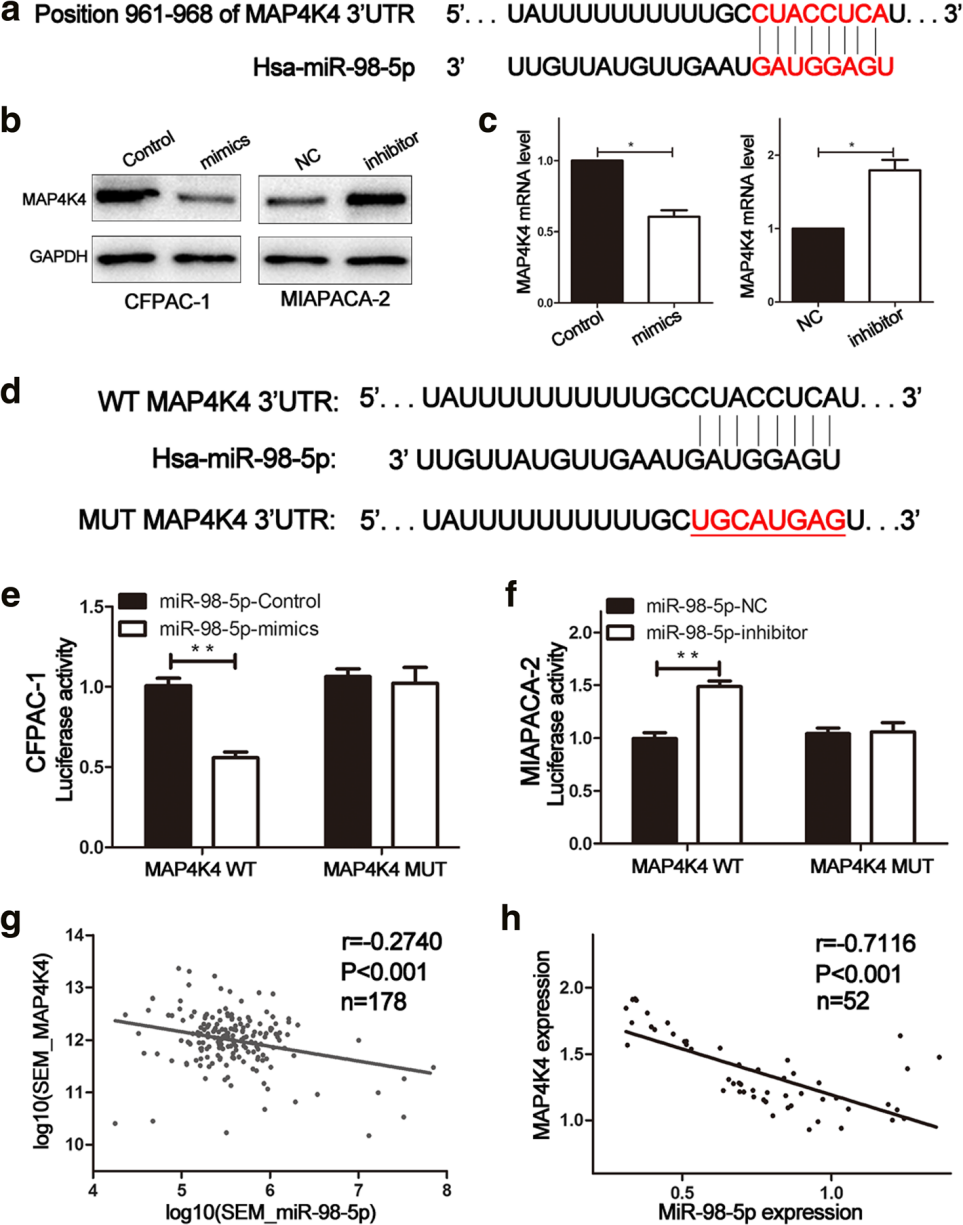
MAP4K4 is a novel target of miR-98-5p. **a** MAP4K4 was predicted as a direct target of miR-98-5p. **b** The expression of MAP4K4 protein in PDAC cells after miR-98-5p alteration by western blot. **c** The expression of MAP4K4 mRNA in PDAC cells after miR-98-5p alteration by qRT-PCR. **d** Luciferase reporter assay was employed to confirm that miR-98-5p directly bound to the 3’UTR region of MAP4K4. **e** Luciferase activity was analyzed in CFPAC-1 cells co-transfected with miR-98-5p-mimics or control with MAP4K4-WT or MAP4K4-MUT. **f** Luciferase activity was detected in MIAPACA-2 cells co-transfected with miR-98-5p-inhibitor or negative control with MAP4K4-WT or MAP4K4-MUT. **g, h** Negative correlation between the expression levels of miR-98-5p and MAP4K4 in PDAC tissues. *P < 0.05, **P < 0.01. The data expressed as the mean ± SD. All experiments were repeated at least three times

TargetScan (http://www.targetscan.org/) and PicTar (http://pistar.mdc-berlin.de/) were employed to identify the target genes of miR-98-5p. Bioinformatics analysis revealed MAP4K4, a well-known tumor oncogene associated with malignant progression in various carcinomas, to be a potential target of miR-98-5p (Fig. 4a).

As shown in Fig. 4b-c, miR-98-5p negatively regulated MAP4K4 expression at both mRNA and protein levels. The role of MAP4K4 as a direct target of miR-98-5p was proved by dual luciferase reporter assay. Wild-type and mutant-type MAP4K4 3’-UTR sequences (MAP4K4-WT and MAP4K4-MUT) were cloned into the pGL3 reporter vector (Fig. 4d). Co-transfection with the MAP4K4-WT and miR-98-5p-mimics in CFPAC-1 cells significantly resulted in decreased luciferase activity compared with that in the control group (Fig. 4e). In contrast, the luciferase activity was increased when co-transfection with the MAP4K4-WT and miR-98-5p-inhibitor in MIAPACA-2 cells (Fig. 4f). Nevertheless, there were no significant differences following co-transfection with MAP4K4-MUT and miR-98-5p-mimics or miR-98-5p-inhibitor (Fig. 4e-f). Furthermore, we discovered that the expression level of miR-98-5p was inversely associated with MAP4K4 expression in 52 paired PDAC specimens (Fig. 4h). The same results were also obtained based on TCGA (Fig. 4g). These results indicated that MAP4K4 was a putative target gene of miR-98-5p in PDAC, which was consistent with our hypothesis.

### MAP4K4 is overexpressed in PDAC tissues and cell lines

**Fig. 5:**
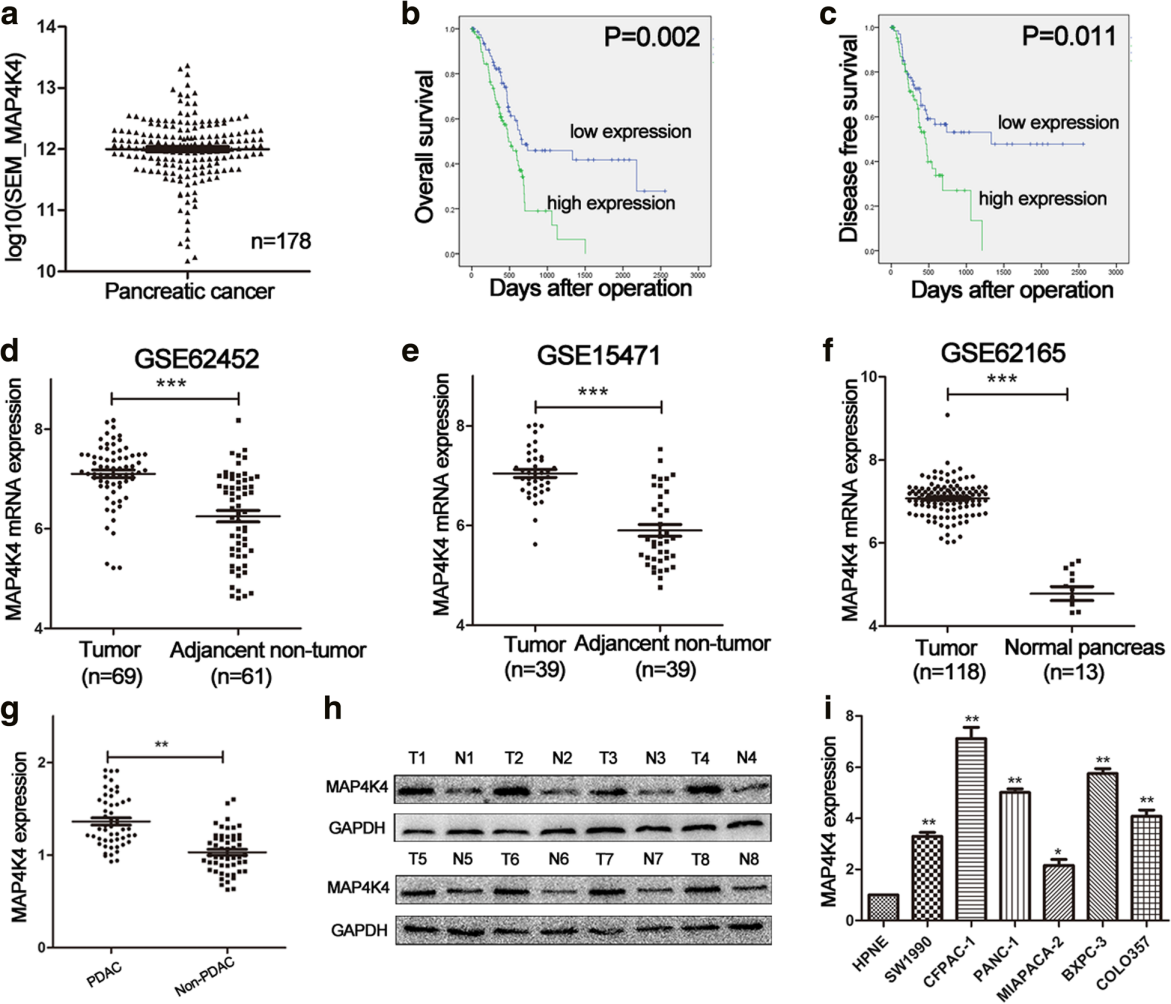
MAP4K4 is upregulated in PDAC tissues and cell lines. **a** MAP4K4 mRNA expression in 178 PDAC samples obtained from TCGA was shown. **b, c** Overexpression of MAP4K4 was associated with poorer prognosis of PDAC. **d-f** Higher expression of MAP4K4 was found in PDAC tissues than that in non-cancerous tissues based on GEO (GSE62452, GSE15471, GSE62165). **g** Higher expression of MAP4K4 was observed in PDAC tissues by qRT-PCR. **h** MAP4K4 protein level was detected using western blot in eight paired of PDAC tissues. **i** The expression of MAP4K4 in different PDAC cell lines was shown. *P < 0.05, **P < 0.01, ***P < 0.001. The data expressed as the mean ± SD. All experiments were repeated at least three times

MAP4K4 mRNA was generally expressed in PDAC tissues (*n* = 178) based on TCGA (Fig. 5a). As shown in Fig. 5d-f, MAP4K4 was significantly upregulated in pancreatic cancerous tissues compared with paired non-tumorous or normal pancreas tissues based on three GEO datasets (GSE62452, GSE15471 and GSE62165). Moreover, analysis from TCGA showed that higher expression of MAP4K4 predicted shorter overall survival time and disease-free survival time by dividing all patients based on the median value of MAP4K4 expression (Fig. 5b-c).

The expression of MAP4K4 was detected in 52 paired PDAC specimens by qRT-PCR. Consistent with GEO datasets, MAP4K4 was higher in PDAC tissues (Fig. 5g). We then detected the protein expression of MAP4K4 in 8 randomly selected pairs of PDAC specimens and adjacent non-cancerous tissues by western blot. As shown in Fig. 5h, MAP4K4 was elevated in PDAC tissues than that in the matched non-cancerous tissues. Similarly, qRT-PCR revealed higher MAP4K4 expression in PDAC cell lines compared with that in HPNE cells (Fig. 5i). Furthermore, we analyzed the association between MAP4K4 expression levels and clinicopathological features. As shown in Table 1, MAP4K4 expression levels showed a highly positive correlation with tumor size, TNM stage and lymph node metastasis in PDAC patients. These observations indicated that MAP4K4 was frequently overexpressed in PDAC.

### MiR-98-5p inhibits proliferation, migration and invasion in PDAC cells by targeting MAP4K4

**Fig. 6:**
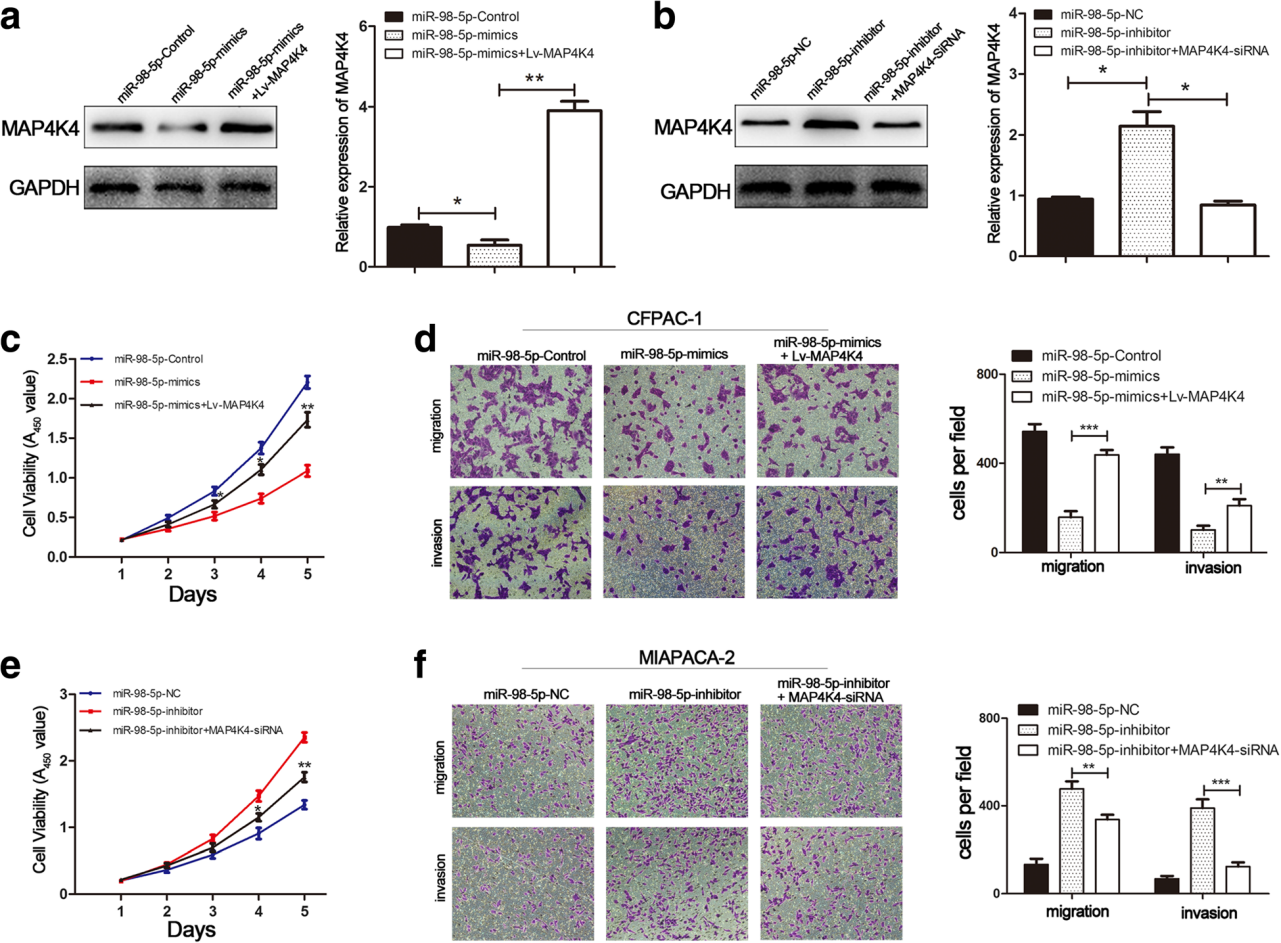
miR-98-5p inhibits PDAC cell proliferation, migration and invasion by targeting MAP4K4. **a, b** Western blot and qRT-PCR were used to determine the expression levels of MAP4K4. **c, e** CCK-8 was used to determine the cell proliferation in different groups. **d, f** The migration and invasion of cells in different groups were investigated by transwell (magnification,100 ×). *P < 0.05, **P < 0.01. The data expressed as the mean ± SD. The data came from at least three independent experiments

We proved that ectopic expression of miR-98-5p suppressed proliferation, migration and invasion and inhibited the MAP4K4 expression by degrading mRNA. Conversely, miR-98-5p knockdown had the opposite effect, leading to elevated protein and mRNA expression of MAP4K4. To further confirm that the effects of miR-98-5p on proliferation, migration and invasion in PDAC cells were mediated by regulation of MAP4K4, we respectively overexpressed MAP4K4 in CFPAC-1/miR-98-5p-mimics cells and silenced endogenous MAP4K4 in MIAPACA-2/miR-98-5p-inhibitor cells (Fig. 6a-b).The results elucidated that ectopic MAP4K4 expression effectively reversed the inhibition of proliferation, migration and invasion induced by miR-98-5p overexpression (Fig. 6c-d). Similarly, MAP4K4 knockdown significantly reversed the promotion effects of proliferation, migration and invasion induced by miR-98-5p depletion (Fig. 6e-f). These investigations were consistent with our hypothesis that miR-98-5p inhibited PDAC cell proliferation, migration and invasion by targeting MAP4K4.

### MAPK/ERK pathway is essential for miR-98-5p in PDAC

**Fig. 7:**
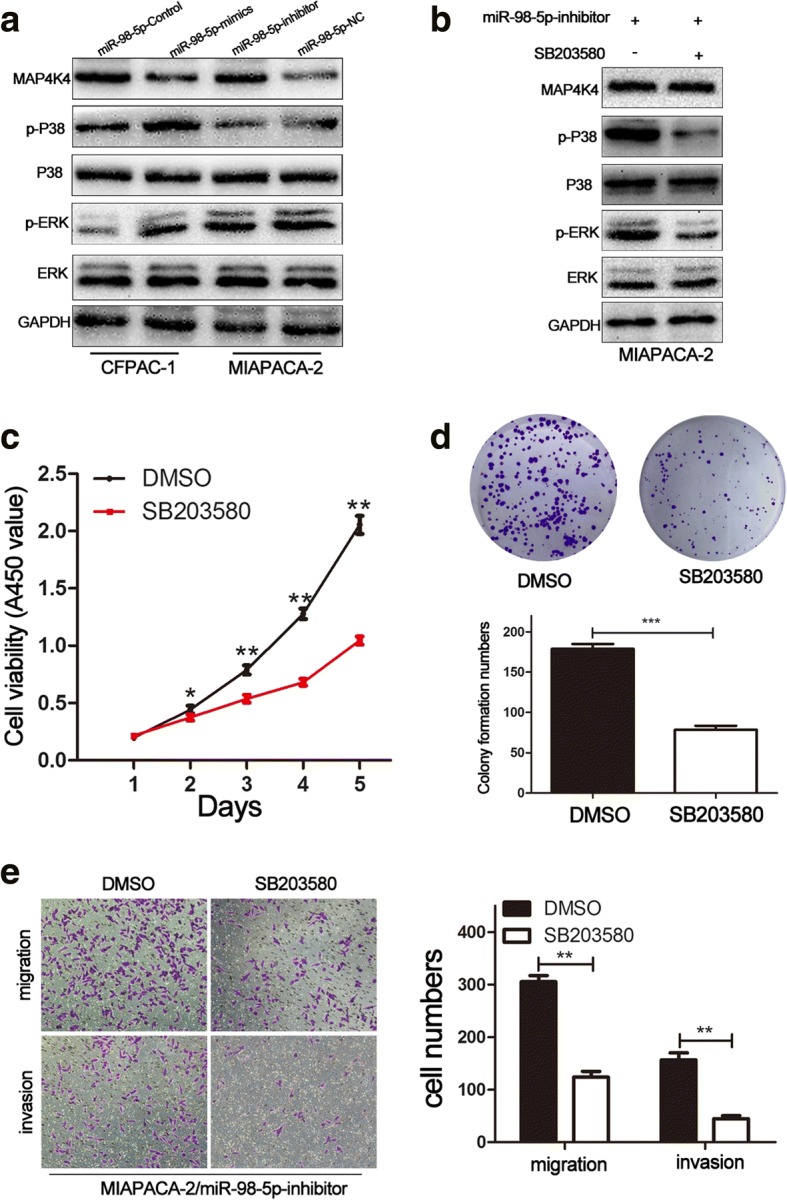
MAPK/ERK pathway is essential for miR-98-5p in PDAC. **a** The expression of MAPK/ERK signaling was evaluated by western blot. **b** MAPK/ERK signaling in MIAPACA-2/miR-98-5p-mimics cells treated with SB203580 or DMSO was measured by western blot. **c, d** The proliferation in PDAC cells following SB203580 treatment was detected by CCK-8 and colony formation assays. **e** The migration and invasion in PDAC cells following SB203580 treatment were determined by transwell. *P < 0.05, **P < 0.01. The data expressed as the mean ± SD. The data came from at least three independent experiments

Considering that MAP4K4 overexpression accelerated malignant progression by activation of the MAPK/ERK signaling pathway in multiple cancers [18, 24], we investigated the role of miR-98-5p in the regulation of PDAC cell proliferation, migration and invasion by detecting MAPK/ERK signaling. As shown in Fig. 7a, miR-98-5p overexpression significantly decreased the expression of phosphorylated p38 and ERK, whereas miR-98-5p knockdown increased in PDAC cells. Nevertheless, the total amount of p38 and ERK showed no differences (Fig. 7a). Furthermore, MIAPACA-2/miR-98-5p-inhibitor cells were treated with the SB203580, a specific inhibitor of MAPK/ERK pathway (Fig. 7b). As expected, SB203580 treatment significantly inhibited PDAC cell proliferation, migration and invasion (Fig. 7c-e). Thus, these results confirmed the involvement of the MAPK/ERK signaling in the promotion of PDAC cell proliferation, migration and invasion by miR-98-5p.

### MiR-98-5p impairs tumorigenicity and metastasis in vivo

**Fig. 8:**
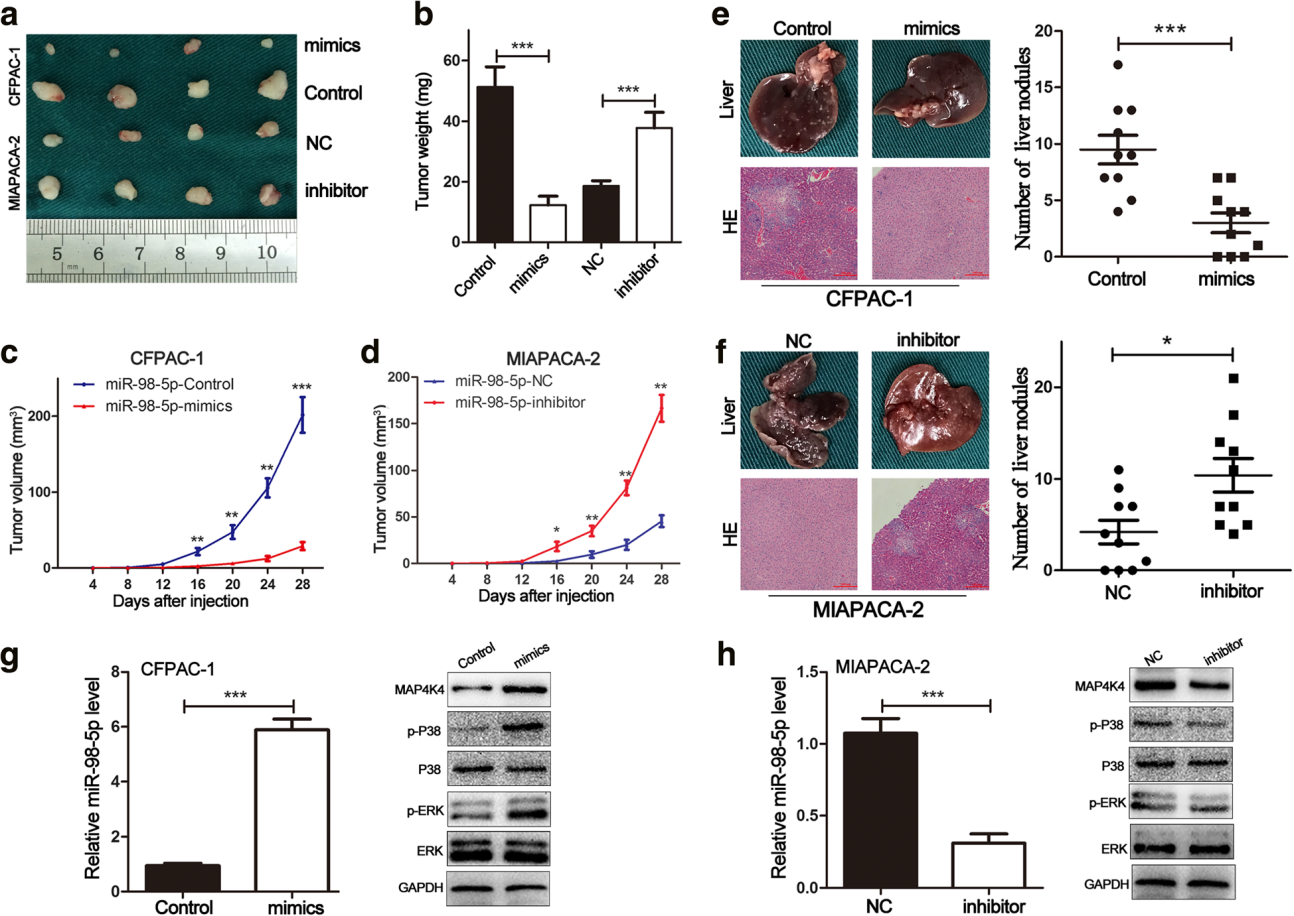
miR-98-5p impairs tumorigenicity and metastasis in vivo. **a** Photograph of tumors obtained from the different groups of nude mice transfected with miR-98-5p-mimics and miR-98-5p-inhibitor, respectively. **b** Tumor weight was calculated. **c, d** Growth curve of tumor volume was calculated. **e, f** Representative images of the livers and its HE staining in different groups were shown (Scale bar, 100 μm). The number of liver metastatic nodules was calculated. **g, h** The expression levels of miR-98-5p, MAP4K4 and MAPK/ERK signaling in the subcutaneous tumors that were transfected with miR-98-5p-mimics and miR-98-5p-inhibitor were explored by miRNA qRT-PCR, qRT-PCR and western blot. *P < 0.05, **P < 0.01, ***P < 0.001. The data expressed as the mean ± SD

To extend our observations, we evaluated whether miR-98-5p influenced the tumorigenesis and metastasis of PDAC cells in vivo. The subcutaneous xenotransplantation model was performed to investigate the effects of miR-98-5p on tumorigenicity. As shown in Fig. 8a-d, the volume and weight of the tumors in the miR-98-5p-mimics group were increased compared with those in the miR-98-5p-control group (CFPAC-1). In contrast, the volume and weight of tumors in the miR-98-5p-inhibitor group were elevated compared with those in the miR-98-5p-NC group (MIAPACA-2). In addition, higher expression of miR-98-5p was observed in the miR-98-5p-mimics group by miRNA qRT-PCR analysis (Fig. 8g). The opposite pattern was discovered in miR-98-5p-inhibitor group than control group (Fig. 8h). Moreover, western blot analysis of the implanted tumor tissues revealed significant downregulation of MAP4K4, p-P38 and p-ERK in the miR-98-5p-mimics group compared with those in the control group. Conversely, the opposite trend was observed in the miR-98-5p-inhibitor group (Fig. 8g-h). However, the total amount of p38 and ERK showed no differences (Fig. 8g-h). Next, the pancreatic tail xenotransplantation model was performed to investigate the effects of miR-98-5p on metastasis. Liver nodule in the miR-98-5p-mimics group was significantly decreased compared with those in the miR-98-5p-control group (Fig. 8e). Conversely, liver nodule in the miR-98-5p-inhibitor group was elevated compared with those in the miR-98-5p-NC group (Fig. 8f). These data suggested that miR-98-5p suppressed proliferation and metastasis of PDAC cells in vivo. The underlying mechanisms might be attributed to downregulation of MAP4K4 and inhibitory of MAPK/ERK signaling pathway.

## Discussion

Tumor proliferation, migration and invasion is a complex and multistep process that is widely regulated by multiple molecules, including miRNAs [5, 25, 26]. MiRNAs have been identified as either oncogenic factors or tumor suppressors depend on the specific functions of targeted mRNAs. A growing body of evidence demonstrated that miRNAs played a vital role in PDAC proliferation and metastasis. Thus, miRNAs have been proposed as potential diagnostic markers and therapeutic targets for PDAC [27–29]. Therefore, increasing insights into the roles and molecular mechanisms of miRNAs during PDAC development are warranted.

MiR-98-5p, a member of let-7 family, has been identified as a tumor suppressor and found to be downregulated in certain malignancies. In HCC, miR-98-5p downregulation stimulated malignant progression through several posttranslational targets, such as IGFBP1, CTHRC1 and SALL4 [10, 30, 31]. MiR-98-5p was significantly increased and promoted cell proliferation and invasion in lung cancer by directly targeting TWIST, PAK1, and ITGB3 [13, 32, 33]. Vinayakumar Siragam et al. reported that miR-98 was involved in the regulation of cell survival, proliferation, tumorigenesis and angiogenesis via targeting ALK4 and MMP11 in breast cancer [34]. Moreover, miR-98-5p played the inhibitory roles in the progression of salivary adenoid cystic carcinoma through impairing PI3K/AKT and MAPK/ERK signaling [35]. In addition, downregulation of miR-98 promoted the migration and invasion in esophageal squamous cell carcinoma (ESCC) through targeting EZH2 [36]. Nevertheless, the function of miR-98-5p and its underlying molecular mechanisms in PDAC remained unclear. In this study, we for the first time showed that miR-98-5p was downregulated in PDAC and was significantly associated with PDAC progression and prognosis. Further gain- and loss-function experiments confirmed that miR-98-5p overexpression inhibited the proliferation, migration and invasion of PDAC cells while miR-98-5p knockdown promoted both in vitro and in vivo. These results indicated that miR-98-5p played a tumor suppressive role in PDAC.

To clarify the mechanisms underlying the effects of miR-98-5p on proliferation, migration and invasion, we predicted putative targets of miR-98-5p in PDAC by bioinformatic analysis. Among the candidate target genes, we focused on MAP4K4, which was located at 2q11.2 in human chrome [37] and contained ~ 1200 amino acids with a molecular mass of ~140KDa [38]. MAP4K4 was initially observed in 1995 as a key kinase in the mating pathway in Saccharomyces cerevisiae and was later discovered to be implicated in several aspects of cell functions and many biological and pathological processes [39]. Accumulating evidence has demonstrated that downregulation of MAP4K4 resulted in induction of apoptosis [18, 22–24, 40], cell cycle arrest [18, 22, 40] and inhibition of cell proliferation [18, 22, 40, 41], migration and invasion [14, 22, 40]. Moreover, negative correlation between MAP4K4 expression and patient prognosis has been reported in multiple types of human cancer, such as CRC [17], lung adenocarcinoma [19] and HCC [18]. Similarly and importantly, there was evidence revealed that MAP4K4 was significantly associated with worse prognosis in patients with Stage II PDAC [20]. Notably, several miRNAs have been certified to regulate MAP4K4 expression in certain cancer. For example, Zhao et al. reported that miR-194 served as a prognostic marker and suppressed cell proliferation by directly targeting MAP4K4 in HCC [41]. Wang et al. found that miR-194 was identified as a tumor suppressor via negative regulation of MAP4K4/c-Jun/MDM2 signaling in CRC [42]. Zhao et al. concluded that miR-141 was downregulated and promoted cell proliferation and invasion via targeting MAP4K4 in pancreatic cancer [40]. In the present study, our analysis elucidated that MAP4K4 was overexpressed in PDAC tissues and cell lines. MAP4K4 expression was also significantly associated with tumor size, TNM stage, lymph node metastasis and prognosis in PDAC patients. Moreover, negative correlation was discovered between expression levels of miR-98-5p and MAP4K4 in PDAC specimens. Furthermore, miR-98-5p negatively regulated MAP4K4 expression at the translational level via binding to a specific target site within the 3’-UTR, which was further proved by luciferase reporter assays. Overexpression of MAP4K4 significantly reversed the effects of the inhibition of proliferation, migration and invasion induced by ectopic miR-98-5p. In addition, siRNA-mediated silence of MAP4K4 impaired the promotion of proliferation, migration and invasion caused by miR-98-5p knockdown. Taken together, the findings of our study indicated that the suppressive effects of miR-98-5p on PDAC were mediated by downregulation of MAP4K4.

Multiple researches confirmed MAP4K4 promoted the malignant progression through several downstream signaling pathways, such as MAPK/ERK1/2, MAPK/JNK, NF-κB, JAK-STAT and Notch [16, 18, 22, 24]. Activation of MAPK/ERK signaling pathway was reported to participate in the development and progression in PDAC [43, 44]. Herein, we discovered that miR-98-5p restrained the activation of MAPK/ERK signaling. The inhibitor of MAPK/ERK pathway abrogated the promoting effects of miR-98-5p knockdown on proliferation, migration and invasion of PDAC cells. Therefore, MAPK/ERK pathway may be involved in the role of miR-98-5p/MAP4K4 axis in PDAC. However, the results of this research do not rule out the possibility that other signaling pathway may also be affected by miR-98-5p.

In conclusion, we find for the first time that miR-98-5p is underexpressed in PDAC tissues and cell lines, and its decreased expression is correlated with malignant clinicopathological features. Furthermore, we confirm that miR-98-5p suppresses proliferation, migration and invasion of PDAC cells via directly targeting MAP4K4-mediated MAPK/ERK pathway. Notably, miR-98-5p downregulation and MAP4K4 upregulation are potential prognostic predictors for the survival of PDAC patients. Taken together, downregulation of miR-98-5p may play an important role in tumor progression and may be a novel prognostic factor and potential therapeutic target for PDAC.

## Conclusions

To conclude, we recognize miR-98-5p underexpression and MAP4K4 overexpression as biomarkers for predicting poor prognosis of PDAC patients. The results of present study provide novel evidence that miR-98-5p inhibits PDAC proliferation and metastasis via targeting MAP4K4 and its downstream MAPK/ERK signaling pathway. This finding will improve understanding of mechanism involved in cancer progression and provide novel targets for the molecular treatment of PDAC.

## Additional file

Additional file 1: **Table S1.** All primers involved in this research (5′-3′). (DOCX 13 kb)

## Abbreviations

CCK-8: Cell counting kit 8
CRC: Colorectal cancer
DMEM: Dulbecco’s Modified Eagle’s Medium
FBS: fetal bovine serum
GEO: Gene Expression Omnibus
HCC: Hepatocellular carcinoma
MAP4K4: Mitogen-activated protein 4 kinase 4
PBS: Phosphate buffered solution
PCR: Polymerase chain reaction
PDAC: Pancreatic ductal carcinoma
PVDF: Polyvinylidene difluoride
SDS-PAGE: Sodium dodecyl sulfate-polyacrylamide gel electrophoresis
TCGA: The Cancer Genome Atlas
