# Supplementary material for: Downregulated miR-98-5p promotes PDAC proliferation and metastasis by reversely regulating MAP4K4
Source: J Exp Clin Cancer Res. 2018 Jul 3;37:130. doi: 10.1186/s13046-018-0807-2 (PMC6029016; doi:10.1186/s13046-018-0807-2)
Supplement: Supplementary file 1 — Table S1. All primers involved in this research (5′-3′). (DOCX 13 kb) [file 13046_2018_807_MOESM1_ESM.docx]

**Additional file 1**

**Table S1: All primers involved in this research (5’-3’)**

| GAPDH forward | CTGGGCTACACTGAGCACC |
| --- | --- |
| GAPDH reverse | AAGTGGTCGTTGAGGGCAATG |
| MAP4K4 forward | GACTCCCCTGCAAAAAGTCTG |
| MAP4K4 reverse | GTCCATAGGTGCCATTTCCAA |
| hsa-miR-98-5p forward | TGAGGTAGTAGTTTGTGCTGTT |
| hsa-miR universal | GCGAGCACAGAATTAATACGAC |
| U6 forward | CTCGCTTCGGCAGCACA |
| U6 reverse | AACGCTTCACGAATTTGCGT |
